# A continuous process of creating responsiveness among healthcare workers results in maintaining good lab practices & increasing awareness regarding safe & hygienic work environment

**DOI:** 10.1186/2047-2994-4-S1-P88

**Published:** 2015-06-16

**Authors:** A-U- Kabeer, SA Muhammad, K Minhas

**Affiliations:** 1 Clinical Laboratory-Histopathology, Aga Khan University Hospital, Karachi, Pakistan

## Introduction

In early 2013, the section of Histopathology of Clinical Laboratory, The Aga Khan University & Hospital was facing the minimal safe practices among technical staff including Improper Hand Hygiene practices, Inadequate Decontamination procedures & Compromised Handling of Biological Spill management. Hence Initiation of Laboratory Staff Awareness Program appeared very productive in improvement of staff routine safe laboratory practices even under stressed & hectic work environment.

## Objectives

The background is that we had encountered number of observations in periodic Safety Inspections regarding Hand Hygiene practices, decontamination procedures & handling of biological spill. Hence in compliance to maintain a safe work environment the laboratory was urged to initiate the Staff Awareness Sessions & Quiz on monthly basis to achieve improvement.

## Methods

In this audit 50 technical staff undergone with assessment in two categories i.e. Awareness; assessed by quarterly quiz and Practices; assessed by direct observation and are evaluated in criteria of Satisfactory, Good & Excellent. The data of two years 2013 & 2014 is analyzed & compared in criteria of improvement from minimal or satisfactory level which is thereby improved up to the level of excellence in staff awareness and routine practices.

## Results

In 2013 hand hygiene was Satisfactory among 86% of staff while by 2014 60% of staff were following an excellent level of hand hygiene.

In 2013 Decontamination practice was Satisfactory among 89 % of staff while by 2014 59% of staff were practicing the excellent level of decontamination.

In 2013 Biological Spill Management was Satisfactory among 96 % of staff while by 2014 it is improved among 68% of staff up to the level of excellent.

## Conclusion

On the basis of above results the achievement in improvement up to the level of excellent observed in 60% of staff for Hand Hygiene, 59% of staff for following proper Decontamination procedure and 68% of staff for Biological Spill Management regarding awareness and implementation in routine practices.

## Disclosure of interest

None declared.

